# Establishment of a Han Chinese-specific pharmacogenetic-guided warfarin dosing algorithm

**DOI:** 10.1097/MD.0000000000012178

**Published:** 2018-09-07

**Authors:** Lin Pei, Xiaoyi Tian, Yan Long, Wenhui Nan, Mei Jia, Rui Qiao, Jie Zhang

**Affiliations:** a The Department of Laboratory Medicine, Peking University Third Hospital; b The Department of Clinical Laboratory Center, Beijing Children's Hospital, Capital Medical University, National Center for Children's Health; c The Department of Laboratory Medicine, Peking University People's Hospital, Beijing, PR China.

**Keywords:** algorithm, CYP2C19, CYP4F2, VKORC1, warfarin

## Abstract

Warfarin is the most common oral anticoagulant. Because of a narrow therapeutic range, interindividual differences in drug responses, and the risk of bleeding, there are many challenges in using warfarin. We need to predict the warfarin maintenance dose. However, ethnic-specific algorithms may be required, and some Chinese algorithms do not perform adequately. Therefore, we aimed to establish a Han Chinese appropriate algorithm.

We recruited a study group consisting of 361 Han Chinese patients receiving warfarin treatment who had heart valve replacements. Genotyping of 38 single nucleotide polymorphisms (SNPs) in 13 candidate genes was carried out using the MassARRAY. In the derivation cohort, a multiple linear regression model was constructed to predict the warfarin dosage. We evaluated the accuracy of our algorithm in the validation cohort and compared it with the other 5 algorithms based on Han Chinese and other races.

We established a Han Chinese-specific pharmacogenetic-guided warfarin dosing algorithm. Warfarin maintenance dosage (mg/day) = 1.787 − 0.023 × (*Age*) + 1.151 × (*BSA* [m^2^]) + 0.917 × (*VKORC1 AG*) + 4.619 × (*VKORC1 GG*) + 0.595 × (*CYP4F2 TT*) + 0.707 × (*CYP2C19 CC*). It explained 58.3% of the variance in warfarin doses in Han Chinese patients and was superior to the other 5 algorithms. The ability of the 6 algorithms which estimate the required dose correctly was tested. Our model had a mean absolute error of 0.74 mg/day, the other 5 models have mean absolute error of 0.81 mg/day,1.05 mg/day, 1.24 mg/day, 1.18 mg/day, and 0.85 mg/day, respectively. Our model had a mean percentage error of 26.9%, the other 5 models have the mean percentage error of 27.7%, 27.2%, 52.3%, 45.7%, and 29.3%, respectively.

Physicians can not adopt algorithm from other race directly to predict warfarin dose in patients with heart valve replacements, they should establish a new algorithm or adjust another algorithm to fit their patients. The algorithm established in this study has the potential to assist physicians in determining warfarin doses that are close to the appropriate doses.

## 1 Introduction

Warfarin is the most common oral anticoagulant. Because of a narrow therapeutic range, interindividual differences in drug responses, and the risk of bleeding, there are many challenges in using warfarin.

Some pharmacogenetics-based algorithms^[1–5]^ integrating patients’ demographic data and genotypes have been developed for predicting the dosage of warfarin. Ethnic variations may result in differential warfarin efficacy and affect the average dose required to maintain therapeutic anticoagulation.^[6]^ However, the performances of some Han Chinese algorithms^[2,3]^ were not adequate.

In this study, we chose and genotyped 38 single nucleotide polymorphisms (SNPs) in 13 candidate genes involved in the biotransformation and mode of action of warfarin. We aimed to investigate the effect of these SNPs on the personalized variability of warfarin dose requirements in Han Chinese patients.

## 2 Methods

### 2.1 Ethics Statement

The study protocol was reviewed and approved by the Ethics Committee of Peking University People's Hospital, Beijing, China and conducted in accordance with the Declaration of Helsinki Principles (revised in 1983)

### 2.2 Study design

**Figure 1:**
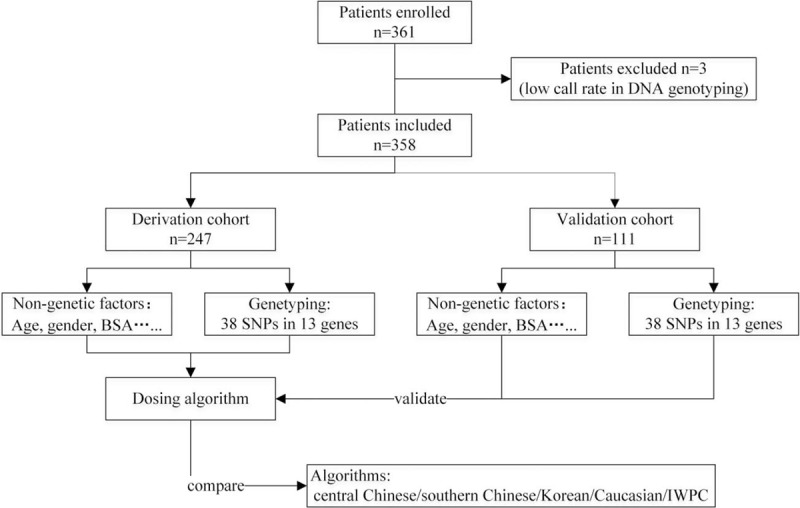
Flow diagram of the study. BSA = body surface area, IWPC = the International Warfarin Pharmacogenetics Consortium, SNP = single-nucleotide polymorphism.

We recruited patients with heart valve replacements. Firstly, the associations between the 38 SNPs and the stable warfarin maintenance dosage were evaluated. Secondly, based on genotypes which associated with the warfarin maintenance dosage, a model integrating patients’ genetic and nongenetic factors was established for predicting the dosage in the derivation cohort (70% of the entire cohort). Thirdly, we evaluated the accuracy of our algorithm in the validation cohort (30% of the entire cohort) and compared the results with those of the other 5 models based on Central Chinese,^[2]^ Southern Chinese,^[3]^ Korean,^[5]^ Caucasian,^[4]^ and a mixed population (International Warfarin Pharmacogenetic Consortium [IWPC]).^[1]^Figure 1 is a flow diagram of the study.

### 2.3 Human subjects

We recruited 361 ethnic Han Chinese patients whose warfarin maintenance dosages were stable from September 2014 to March 2015. All participants in the study had received heart valve replacements at Fuwai Hospital or Peking University People's Hospital and received regular anticoagulation treatment after that.

*Inclusion criteria*: ≥18 years old; received warfarin treatment for at least 3 months; in a stable condition with a constant warfarin dosage for at least 1 week; with the international normalized ratio (INR) values within the range of 2.0 to 3.0 more than once after hospitalization.

*Exclusion criteria*: Patients with kidney or liver dysfunctions, malignant tumors, autoimmune diseases, or infections were excluded from the study.

We used patient interviews and a review of medical records by trained clinicians to collect data on the following clinical parameters: sex, age, height, weight, concomitant diseases, concurrent interacting medications, smoking habits, and alcohol consumption.

### 2.4 Blood sampling

We collected blood samples (3 mL) from each patient and placed them in sodium citrate tubes for deoxyribonucleic acid (DNA) analysis and prothrombin time (PT)-INR determination. The INR was measured in the plasma immediately after collection. The cell pellets were used for DNA extraction and stored at −70 °C condition.

### 2.5 INR and clinical data

The patient data were collected at regular interviews, and the demographic data (sex, age, height, weight, smoking and drinking habits, and stable warfarin maintenance dose) for the study group are listed in Table 1.

**Table 1:**
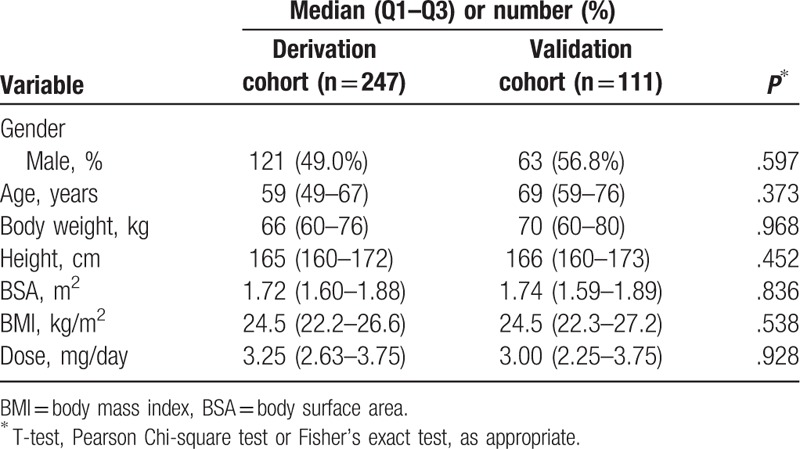
Characteristics of the study population.

The body surface area (BSA) was calculated with the height and weight using the following equation: BSA (m^2^) = 0.0061 × height (cm) + 0.0128 × weight (kg) − 0.1529. The body mass index (BMI) was calculated as follows: weight (kg)/[height (m)]^2^. The mean daily stable dosage was obtained from records over a continuous period of at least 3 months in which the INR was in the range of 2.0 to 3.0. The PT-INR was measured using an ACL TOP 700 instrument (Instrumentation Laboratory, Lexington, MA).

### 2.6 Genotyping

We prepared the genomic DNA with DNA blood kits (TIANGEN) according to the recommendations of the manufacturer, and genotyped 38 SNPs in 13 genes (vitamin K epoxide reductase complex subunit 1 [VKORC1], cytochrome P450 2C9 [CYP2C9], epoxide hydrolase 1 [EPHX1], cytochrome P450 2C19 [CYP2C19], calumenin [CALU], cytochrome P450 4F2 [CYP4F2], cytochrome P450 2C18 [CYP2C18], cytochrome P450 3A4 [CYP3A4], protein C receptor [PROCR], myelin protein zero (MPZ), syntaxin 4A (STX4A), ATP binding cassette subfamily b member 1 [ABCB1], and gamma-glutamyl carboxylase [GGCX]) using the MassARRAY high-throughput DNA analysis system with matrix-assisted laser desorption/ionization time-of-flight mass spectrometry (MALDI-TOF MS, Agena Bioscience, Inc., San Diego, CA). Primers were designed using Assay Design Suite (version 2.0, Agena Bioscience, Inc., San Diego, CA). The SNPs were genotyped using iPLEX Gold technology (Agena Bioscience Inc., San Diego, CA) followed by automated data analysis using the MassARRAY Typer software version 4.0. Three samples were removed due to failed genotyping.

### 2.7 Linear regression modeling

The relationship between subject characteristics and warfarin dose was explored. Firstly, a forward selection procedure (*P < *.2) was used, and in this step, all the variables were added to the model one at a time. Secondly, a backward stepwise selection was used to refine the model with a threshold *P*-value = .05 to include the variables in the final predictive model.

### 2.8 Validation of the algorithm for predicting stable warfarin maintenance dosage

The clinical significance of the algorithm was estimated by determining the percentage of patients for whom the actual warfarin dose was well predicted. A previously described method was used for this analysis.^[1,7]^ A predicted warfarin maintenance dosage within 20% of the actual dosage was considered a successful prediction (ideal dose) while predicted doses below and above the actual dosages by > 20% were considered underestimations overestimations, respectively. The use of 20% as the cutoff limit was consistent with other dosing algorithms.^[8]^ Using the same definition of the “ideal dose” enabled the results of the present analysis to be compared with those of other studies.

### 2.9 Comparison of present model with 5 other algorithms based on Han Chinese and other races

To determine the efficiency of our algorithm, we selected 5 algorithms based on Han Chinese,^[2,3]^ Korean,^[5]^ Caucasians,^[4]^ and mixed race1 parameters to estimate the variability in required dosage. The predicted dosage using the algorithm was plotted versus the actual warfarin maintenance dosage, and a linear regression curve was fitted. The adjusted coefficient of determination (R^2^ statistic), mean absolute error, mean percentage error, and the slope and intercept of the regression line were used to estimate the accuracy of the models.

### 2.10 Statistical analysis

The continuous and categorical variables were expressed as medians (Q1–Q3) and counts (percentages), respectively. The differences between the derivation and the validation cohorts were evaluated using the chi-square test. The univariate association between each potential predictor and the warfarin maintenance dosage was evaluated using linear regression analysis. The Hardy–Weinberg equilibrium (HWE) of genotyping data in the deviation cohort were assessed using the chi-squared test, and the association with the warfarin maintenance dosage was analyzed using the Spearman correlation analysis. We selected potential predictors as candidate variables for the model with a *P < *.20 using stepwise multiple regression in the derivation cohort. Our algorithm was validated using the Pearson correlation analysis in the validation cohort, and all the data were processed using the statistical package for the social sciences (SPSS, ver. 19.0, SPSS Science, Chicago, IL).

## 3 Results

### 3.1 Patient characteristics

A total of 361 patients were initially enrolled. Because the call rate of DNA genotyping was low, 3 patients were excluded. Figure 1 shows the characteristics of the 247 and 111 patients enrolled in the derivation and validation cohorts, respectively. The patients enrolled early were allocated to the derivation cohort. Table 1 shows the demographic and clinical features of all the participants, and no significant difference was observed in age, sex, body height, weight, BSA, and the warfarin maintenance dose between the 2 groups (*P > *.05).

### 3.2 Establishment of warfarin dosing algorithm

Table 2 shows the genotyping results. The allele frequencies of all genes in this study were in accordance with the HWE. The minor allele frequencies (MAF) of all SNPs in this study were in accordance with those observed in other studies in Chinese populations, but the MAF of some SNPs (e.g., VKORC1 rs9923231) were not in accordance with the global MAF.

**Table 2:**
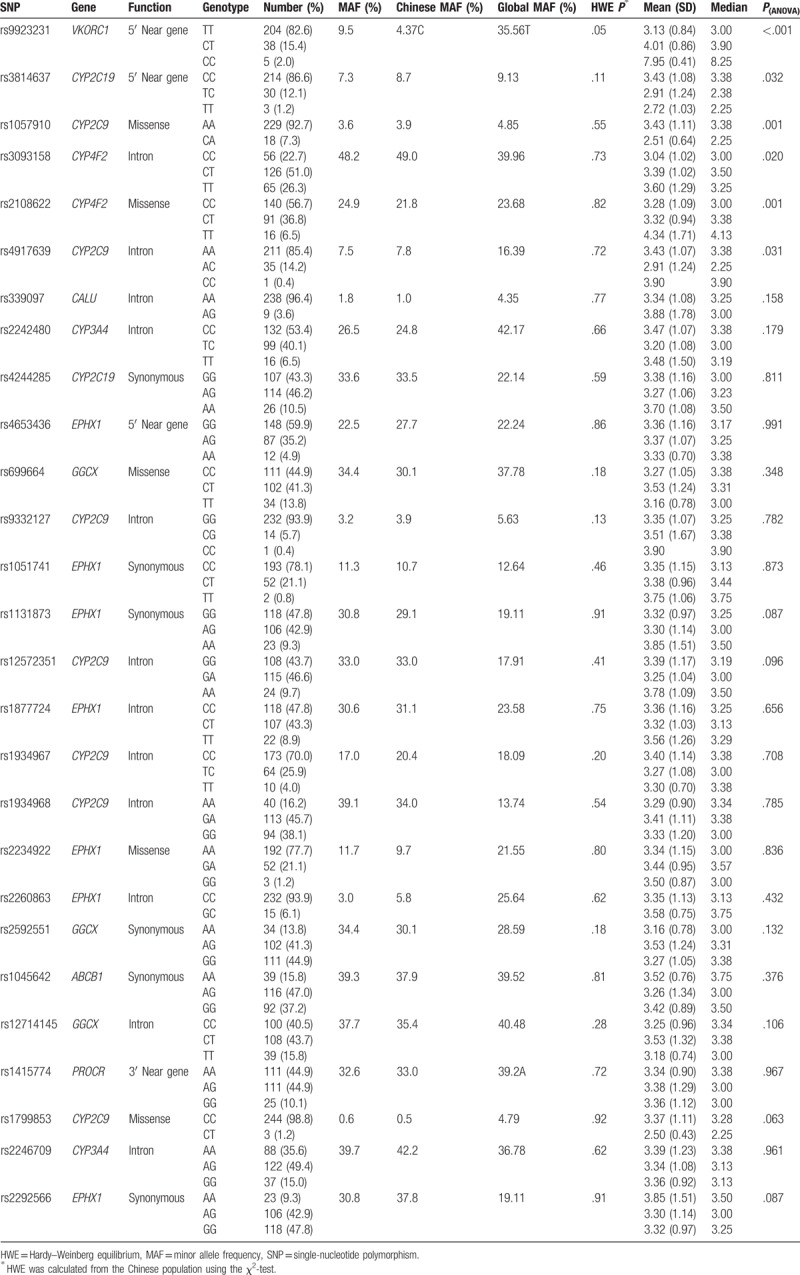
Association of candidate SNPs with warfarin maintenance dose in the derivation cohort.

The genotyping results showed that the polymorphisms of CYP2C9∗5 (rs28371686), CYP2C9∗6 (rs9332131), and GGCX (rs11676382) were absent in our Han Chinese derivation cohort. Furthermore, 6 of the 38 SNPs (VKORC1 rs9923231, CYP2C9∗3 rs1057910, CYP2C9 rs4917639, CYP2C19 rs3814637, CYP4F2 rs2108622, and CYP4F2 rs3093158) exhibited a significant association with warfarin maintenance dosage (Table 2). The maintenance dosage was 154%, 28%, and 43% higher in VKORC1 rs9923231 GG, AG and G allele, respectively than it was in VKORC1 rs9923231 AA patients. The maintenance dosage was 32.3%, 1.2%, and 3.5% higher in CYP4F2 rs2108622 TT, CT, and T allele, respectively, than it was in CYP4F2 CC patients.

The correlation between nongenetic factors (age, sex, height, weight, BMI, BSA, and smoking) and the stable warfarin dosage was analyzed in the derivation cohort. The factors with a linear regression *P < *.20 were sex, age, BSA, and smoking. After including 13 genetic factors with a *P < *.20, 17 factors were taken into the stepwise regression analysis. Finally, only 5 factors were included in the final regression model. The impact factors included (*R*^2^ = 58.3%) in the final model are displayed in Table 3. In this regression model, VKORC1 and CYP2C19 contributed most to the interindividual variability in the warfarin maintenance dosage, accounting for 42.9% and 4.3%, respectively. CYP4F2 could explain approximately 1.6% of individual differences in daily stable dosage, which was less than the results above. Age and BSA contributed most (5.2% and 4.3%, respectively) to the interindividual variability of the nongenetic factors. To obtain a patient's stable maintenance dosage using our algorithm, a doctor would calculate using the following algorithm:

**Table 3:**
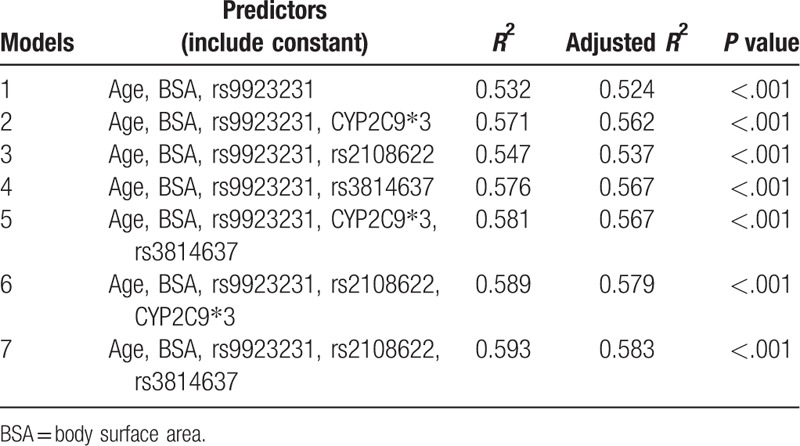
Multiple linear regression for modeling daily warfarin dosage requirements.

Warfarin maintenance dosage (mg/day) = 1.787 − 0.023 × (Age) + 1.151 × (BSA [m^2^]) + 0.917 × (VKORC1 AG) + 4.619 × (VKORC1 GG) + 0.595 × (CYP4F2 TT) + 0.707 × (CYP2C19 CC). The presence and absence of a gene polymorphism are denoted by “1” and “0,” respectively.) Overall, the algorithm explained 58.3% of the interindividual variability in stable warfarin dosages.

### 3.3 Validation of warfarin dosing algorithm

The predicted warfarin maintenance dosage was calculated using our model in the validation cohort (n = 111). We assessed the efficiency of the present algorithm using Pearson correlation analysis and a moderately strong correlation was observed between the predicted and the actual dosages (Pearson *r* = 0.722, *P* < .001).

Moreover, the accuracy of the algorithm in the subgroups according to the warfarin dosage range (Table 4) was evaluated. The result showed that the accuracy of the prediction in the intermediate dose (2–4 mg/day) group was much higher than that of the other 2 groups. Specifically, 65.9%, 21.4%, and 57.6% of the predicted dosages fell within 20% of the actual dosage (ideal dose) in the intermediate-, low-, and high-dose groups, respectively. We found that 42.4% and 78.6% of the predictions were underestimated and overestimated in the high- and low-dose groups, respectively.

**Table 4:**
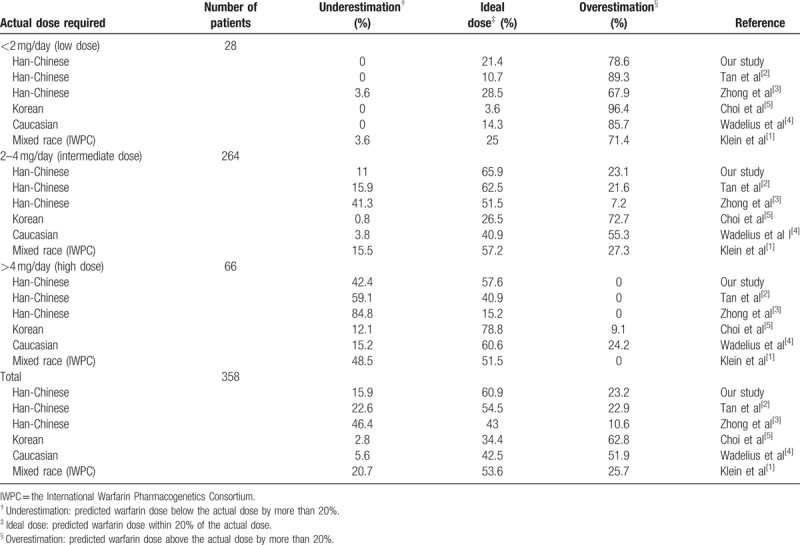
Percentage of patients in the whole cohort with an ideal, underestimated or overestimated dose of warfarin estimates with algorithms derived in different ethnicity.

### 3.4 Comparison of our algorithm with 5 others

**Figure 2:**
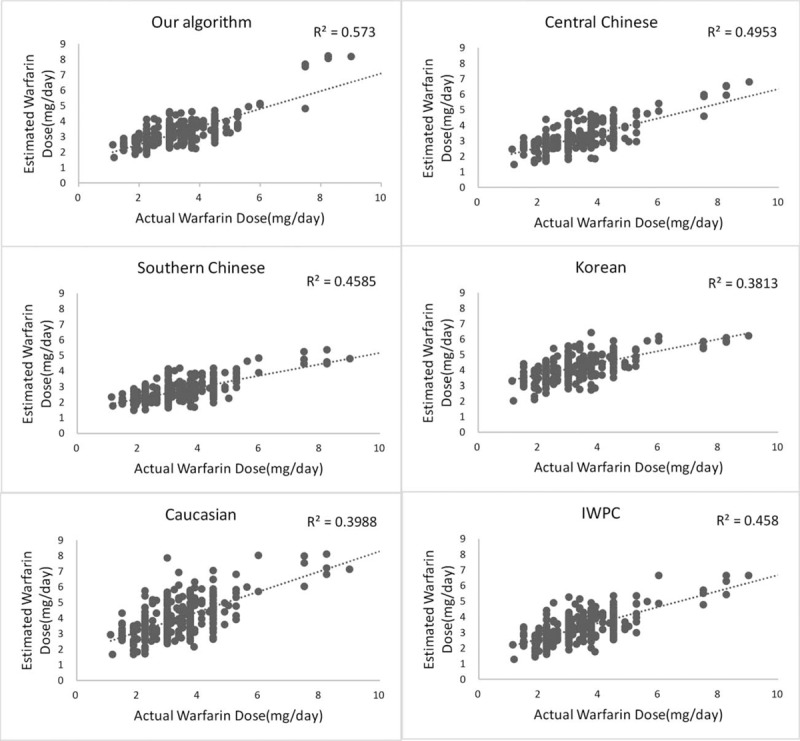
Comparison of the forecasting ability for each algorithm.

We compared our algorithm with 5 other algorithms based on a central Chinese,^[2]^ southern Chinese,^[3]^ Korean,^[5]^ Caucasian,^[4]^ and a mixed population (IWPC).^[1]^ The scatter plots of the predicted against actual warfarin doses for each algorithm are displayed in Figure 2. The scatter plots revealed that the predicted errors varied with dosage. The dosages of more patients, whose actual dosage was < 2 mg/day, were overestimated while dosages of more patients, whose actual dosage was > 4 mg/day, were underestimated.

By the means of other summary statistics shown in Table 5, we can take a deeper insight into the ability of the algorithms to correctly predict the required dose. Our algorithm showed a mean absolute error of 0.74 mg/day and a mean percentage error of 26.9%. Our model displayed a moderately strong correlation between the predicted and the actual dosage (Pearson *r* = 0.757, *P < *.001).

**Table 5:**
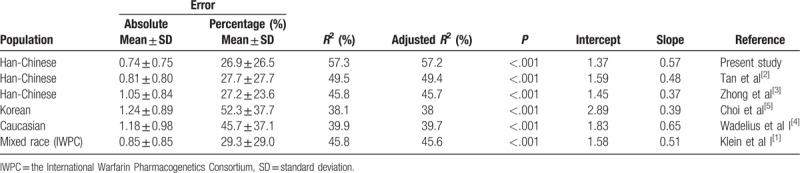
Comparing our algorithm with other 5 algorithms.

We could explain 58.3% of the variability of warfarin maintenance dosage using our algorithm. In the entire cohort, the percentage of patients with an ideal, underestimated, or overestimated warfarin dosage predicted using the 6 algorithms are shown in Table 4. All the algorithms showed higher predictive accuracy for the intermediate-dose group than they did for the low- or high-dose groups. Our algorithm and the central Chinese algorithm displayed better performances for the intermediate-dose group than the other algorithms did. Our algorithm, as well as the Korean and Caucasian algorithms, performed better for the high-dose group than the other algorithms did. Our algorithm, the central Chinese algorithm, and IWPC algorithm performed better for the low-dose group. In the entire cohort, our model had a coefficient value of 0.573 (*P* < .001, Fig. 2) using the Pearson correlation analysis.

## 4 Discussion

A new model has been established to predict the warfarin maintenance dosage, the individualized management of warfarin treatment in Han-Chinese patients with heart valve replacement will be improved.

Among the 38 candidate SNPs, the MAF of VKORC1 rs9923231 varies by ethnicity, with the highest, intermediate, and lowest frequency occurring in Asians (82%–96%),^[9]^ Europeans, African–Americans,^[10]^ respectively. Differences in allele frequency lead to lower stable warfarin maintenance dosage in Asians than in Europeans.^[10]^ In our study, although the MAF of VKORC1 rs9923231 differed from that of global populations (Table 2), the VKORC1 haplotype contributed the most (42.9%) to the warfarin dose.

Several genetic markers have been hypothesized to affect the stable warfarin maintenance dosage including the CYP4F2, CALU, and GGCX^[11–14]^ genes.

However, Lee et al^[15]^ discovered that the CYP4F2 (rs2108622) has little effect on warfarin maintenance dose in Han-Chinese. It was found that the influence of the CYP4F2 rs2108622 genotype varied in different populations,^[16–19]^ and CYP4F2 rs2108622 should be considered before prescribing warfarin. This previous finding was consistent with our results. CYP4F2 is a vitamin K1 oxidase, and rs2108622 is expected to influence vitamin K1 levels, which might explain the influence of the CYP4F2 rs2108622 genotype on warfarin dose.^[20–22]^

In our study, we found that in Han Chinese, the warfarin dose was 32.3%, 1.2%, and 3.5% higher in the CYP4F2 TT, CT, and T alleles, respectively, than it was in CYP4F2 CC patients. The corresponding values in Caucasians were 23.0%, 10.0%, and 11.0%,^[23]^ respectively. CYP4F2 rs2108622 has a small but significant association with stable warfarin dosage.

CYP2C9 is a member of the CYP superfamily of enzymes, which are responsible for the metabolism and elimination of numerous common prescription drugs.^[24]^ The frequency of mutant CYP2C9∗3 rs1057910 is lower in the Han Chinese population than it is in Indians and Caucasians. The distribution of the CYP2C9∗3 genotype in the Han Chinese population significantly is different from that in Africans, Caucasians, and South and West Asians.^[25]^ The univariate analyses revealed that rs1057910 was significantly related to stable warfarin dosage. However, in the backward stepwise multiple regression studies, CYP2C9 rs1057910 was not retained in the final algorithm. The reason for this finding remains to be explored further. Mutant CYP2C9∗2 rs1799853 is uncommon in East Asians including the Han Chinese, Japanese, and Koreans, although it is general in South and West Asians and Caucasians.

There are no CYP2C9 rs9332127 variants in Caucasian individuals, but in the Chinese, it affects the warfarin maintenance dose.^[26–28]^ In our study, rs9332127 was not associated with the warfarin maintenance dosage.

Two nongenetic factors (age and BSA) contributed to the variability of warfarin maintenance dosage, it is consistent with the results of previous studies.

Age is an important variable, and elderly patients display increased sensitivity to warfarin, which has been previously reported.^[29]^ Shepherd et al^[30]^ demonstrated that there was no apparent difference in warfarin pharmacokinetics between younger (20–40 years old, mean 25 years) and older (65–94 years old, mean 82 years) patients. The increased sensitivity to warfarin is possibly caused by a decrease in the activity of the vitamin K redox recycling system.^[29]^

In some studies, sex was included in the final regression models.^[31,32]^ In our study, sex was not significantly associated with warfarin maintenance dosage in the univariate analysis. This phenomenon likely occurred because the BSA, which was larger in men than in women, was included in the model.

Our model explained 58.3% of the variability of stable warfarin dosage, indicating that it has a higher efficiency to predict the stable warfarin dosage than some other Han Chinese algorithms do.

In the linear regression analysis between the actual and predicted dosages, the coefficient of the Pearson correlation analysis in our algorithm was 0.573 (*P < *.001, Fig. 2). Therefore, our algorithm was the most accurate of the 6 algorithms investigated.

Our algorithm had the lowest mean absolute error (0.74 mg/day) and mean percentage error (26.9%). The Korean algorithm had the highest mean absolute error (1.24 mg/day) and mean percentage error (52.3%). The prediction error of our algorithm was the lowest of the 6 algorithms. The mean absolute error statistic measures how close the predicted dose to the actual dose. A slope of 1 and an intercept of 0 indicates no proportional and constant errors, respectively. The intercept of the Korean algorithm was the highest (2.89) of the 6 algorithms, which indicates that the overprediction was more at the low dose. The intercept of our algorithm was the lowest (1.37) of the 6 algorithms.

There are some limitations to our study. Firstly, it was not designed to achieve a target INR value outside the range of 2.0 to 3.0, and in contrast to other studies, it was not included in the algorithm.^[28]^ Secondly, a larger group of independent patients would be required to validate our algorithm. In summary, algorithms derived from other countries are not suitable for Chinese populations, and ethnic-specific warfarin dosing algorithms are required.

## 5 Conclusions

We established a novel algorithm. Furthermore, compared with the algorithms derived from other Han-Chinese-based algorithms, our newly developed model could improve individualized management of warfarin treatment in Han Chinese patients with heart valve replacement.

## Author contributions

**Data curation:** Xiaoyi Tian, Wenhui Nan, Yan Long.

**Formal analysis:** Wenhui Nan.

**Investigation:** Lin Pei, Xiaoyi Tian, Yan Long.

**Methodology:** Lin Pei, Mei Jia, Jie Zhang.

**Project administration:** Jie Zhang.

**Software:** Lin Pei, Rui Qiao.

**Supervision:** Mei Jia, Jie Zhang.

**Writing – original draft:** Lin Pei.

**Writing – review & editing:** Lin Pei.

Lin Pei: 0000-0003-4904-199X
